# The Cross-Sectional Study of attitudes towards risk factors of viral infections transmitted by blood-borne pathogens

**DOI:** 10.1590/1980-220X-REEUSP-2022-0097en

**Published:** 2023-03-31

**Authors:** Sunčica Ivanović, Sanja Trgovčević, Milena Cvetković Jovanović, Biljana Kocić, Suzana Milutinović

**Affiliations:** 1 College of Applied Health Sciences Ćuprija, Public health, Ćuprija, Republic of Serbia.; 2 Institute for emergency medicine in Niš, Ćuprija, Republic of Serbia.; 3 University of Niš, Faculty of Medicine Niš, Narrow scientific field Infectivity and Epidemiology, Ćuprija, Republic of Serbia.

**Keywords:** Occupational Risks, Vaccination, Infections, Education, Delivery of Health Care, Riscos Ocupacionais, Vacinação, Infecções, Educação, Atenção à Saúde, Riesgos Laborales, Vacunación, Infecciones, Educación, Atención a la Salud

## Abstract

**Objective:**

The objective of this paper was threefold: To assess risk factors of blood-borne pathogen exposure and viral infection for employees at their workplace, to spot the differences between groups of respondents without exposure and those exposed to blood-borne infections, and to identify main risk predictors.

**Method:**

The Cross-Sectional Study was conducted, surveying 203 employees, at the Institute for Emergency Medical Services in Serbia, which were eligible to enter the study and surveyed by Previously Developed Questionnaire.

**Results:**

A total of 97.60% of respondents have perceived risk at their workplace, but there were low numbers of HIV, HbcAg, and Anti-HCV testing and poor percent of vaccination for hepatitis B. There were no statistically significant differences between spotted groups of respondents in their attitudes. Three variables were predictors: accidental usedneedle stick injuries (OR = 90.34; 95% CI, 8.79–928.03), contact with the blood of patientsthrough the skin (OR = 176.94; 95% CI, 24.95–1254.61), and the years of service (OR = 0.92; 95% CI, 0.86–1.00).

**Conclusion:**

The significance of this study is that it points to a double risk, because not only health workers are endangered, but also citizens who receive first aid.

## RESUMO

**Objetivo:**

O objetivo deste estudo foi triplo: avaliar os fatores de risco de exposição a patógenos transmitidos pelo sangue e infecções virais para funcionários no local de trabalho, perceber diferenças entre grupos de indivíduos que não foram expostos e aqueles que estavam expostos a infeções transmitidas pelo sangue e identificar os principais preditores de risco.

**Método:**

Foi realizado um estudo transversal, entrevistando 203 funcionários do Instituto de Assistência Médica de Emergência da Sérvia, que cumpriram as condições para fazer parte do estudo e foram entrevistados por meio de um questionário previamente elaborado.

**Resultados:**

Um total de 97,60% dos entrevistados percebeu risco em seu local de trabalho, mas houve um pequeno número de testes de HIV, HbcAg e Anti-HCV e um baixo percentual de vacinação contra hepatite B. Não houve diferenças estatisticamente significativas entre os grupos observados de entrevistados em termos de suas atitudes. Três variáveis foram preditores: lesões acidentais da punção com agulha (OR = 90,34; 95% Cl, 8,79–928,03), contato com o sangue dos pacientes através da pele (OR = 176,94; 95% Cl, 24,95–1254,61) e tempo de serviço (OR = 0,92; 95% Cl, 0,86–1,00).

**Conclusão:**

O significado deste estudo é que indica um duplo risco, tendo em vista que não apenas os profissionais de saúde estão em risco, mas também os cidadãos que recebem primeiros socorros.

## RESUMEN

**Objetivo:**

El objetivo de este trabajo fue triple: estimar los factores del riesgo de la exposición de los patógenos transmitidos por la sangre y infecciones virales para los empleados en el puesto de trabajo, advertir las diferencias de los grupos de los examinados que no fueron expuestos y los que fueron expuestos a las infecciones que se transmiten por la sangre e identificar los mayores predictores del riesgo.

**Método:**

Fue hecho el estudio de la sección, con una encuesta de 203 empleados en la Institución para los primeros auxilios de Serbia, que cumplieron las condiciones para ser la parte del estudio y encuestado a través del cuestionario.

**Resultados:**

Total de 97,60% de los examinados mostró el riesgo en su puesto de trabajo, pero existía el pequeño número de testes a VIH, HbcAg y Anti – HCV y bajo porciento de vacunación contra Hepatitis B. No hubo diferencias estadísticas significantes entre grupos reconocidos de los cuestionados según sus opiniones. Tres variables fueron los predictores: las lesiones de pinchazo con la aguja accidentales (OR = 90,34; 95% CI, 8,79–928,03), contacto con la sangre dentro de la piel (OR = 176,94; 95% CI, 24,95–1254,61) y experiencia laboral (OR = 0,92; 95%CI, 0,86–1,00).

**Conclusión:**

El significado de este estudio es lo que muestra riesgo doble, teniendo en la cuenta que no son amenazados solo los trabajadores sanitarios, sino y los ciudadanos que reciben primeros auxilios.

## INTRODUCTION

Occupational exposure of health workers in the health care organizations needs to assess the blood-borne transmitted viral infections problem primarily through quantifying frequency and risk factors. These assessments are essential for organizing and implementing prevention and control measures. The majority of these infections arise due to the professional exposure of health workers to the risky contact with potentially infectious material^(1)^. Professional exposure to blood is a percutaneous injury (e.g. needle stick injury or cut with a previously used sharp medical object) or contact between the mucous membrane or non-intact skin (e.g. exposed skin that has cracked, or that was abraded or affected by dermatitis) and blood, tissues or other body fluids, which can lead the healthcare worker to be at risk of hepatitis B virus (HBV), hepatitis C virus (HCV), or human immunodeficiency virus (HIV)^(2)^. At least twenty occupational groups are exposed to different pathogens that can be transmitted through blood during work due to exposure to injury by prick, a needle stick injury or cuts made by sharp objects^(3,4)^. World Health Organization (WHO) emphasize that despite improved methods of preventing exposure in the workplace, occupational exposure to blood-borne pathogens will continue to occur, and that 90% of all infections of health workers can be attributed to exposure at work, which causes enormous concern within health establishments and among health care-workers. Especially in today time, while health care workers are fighting against Covid-19^(5)^. Around the world, every year of 35 million health workers, three million are exposed to blood-transmitted diseases, 2 million to HBV and 0.9 millionto the virus hepatitis C (HCV). The consequence can lead to potentially new 70 thousand HBV and 15 thousand HCV infections. Countries in development lead with more than 90% of these infections. The most common cause of these infections were injuries that led to parenteral entry of the agent. What is especially important is that they could have been prevented. After the injury on the needle, from the patient to the healthcare professional, the risk of transmitting HBV ranges from 1–6% if the patient is HBEAG negative, and from 19–40% if the patient is positive with the antigen^(5,6)^.

In Europe, the average incidence for HBV in 2011 was 1.76 per 100 thousand inhabitants, while in Germany, HCV was 6.6/100,000 inhabitants^(7,8)^. Research conducted in the Republic of Serbia, in Vojvodina, shows that among 5,203 respondents of employees in healthcare (most commonly in nurses), more than 30% were injured by a needle, 16% were injury on a sharp object, 38% had skin contact with blood, and almost 14% had contact of mucosa with blood^(7)^.

On average, in Germany, 500,000 injuries with a needle happen to medical workers on a yearly basis, and only 28.7% of the injured are registered and consol a doctor^(7)^.

Research done in Turkey registered from 516 surveyed nurses, 79.7% have been pierced by a needle during their working life, with 68.4% in the last year. These injuries were more frequent in nurses with less than 4 years of work experience and under 24 years of age^(9)^.

Blood-borne infections are a major problem for healthcare professionals as well as the health care system and for policy makers in making important decisions to reduce the risk and infection of blood-borne diseases. This is one of the reasons why high-income countries have established a system for monitoring the exposure of blood and body fluids among health workers^(10)^. These risks include the number and types of contacts with blood, the prevalence of blood-borne infections, and the risk of transmitting the infection after one contact with blood^(11)^. An unplanned and unwanted exposure event at first may not cause significant injuries to the employee and may temporarily hinder them or their work efficiency for 2–3 days, but left unchecked they can become potential risk factors for the occurrence of infectious disease, injury, reduction of work capacity or death^(12)^. Low rates of reporting blood or body fluid exposure among health workers is one of the most important occupational risk factors^(12)^. This low level of reporting also indicates that the lack of a fundamental understanding from health professionals about the importance of reporting contacts with blood, the prevalence of blood-borne infections, and the risk of transmitting the infection after one contact with blood^(13)^. We suggest that there should be an emphasize on the need for medical workers to respect protocols regarding infected blood so as to not become infected/carriers themselves and then transmit the disease to patients to whom they are giving emergency medical attention. The study is of great importance because it includes employees in emergency medical service which are at great risk of getting viral blood infections when saving the lives of endangered individuals.

Therefore the aim of this paper was threefold: To assess risk factors of blood-borne pathogen exposure and viral infection for employees at their workplace, to spot the differences between groups of respondents without exposure and those exposed to blood-borne infections, and to identify main risk predictors.

## METHOD

### Design of Study

A Cross-Sectional Study was conducted through anonymous and voluntary surveying of employees at the Institute for Emergency Medical Services in Niš, Serbia, in 2018.

### Population

They are four Institutes for Emergency Medical Services in Serbia, and 900 employees health care workers. The sample size was calculated via G* Power 3.1.9.7^(14)^. Effect size observed in this study was f = 0.50 and for a test power and confidence level of 95%, 124 individuals were required. Institute for Emergency Medical Services in Niš at the time of conducting the research, had 247 employed workers, of which 69 were doctors, 78 medium medical staff, and 100 auxiliary medical and non-medical staff. Total of 203 questionnaires were collected. Of this number 80 questionnaires did not enter the study because respondents did not adequately fill the questionnaire, or did not provide it (due to annual rest or sick leave) were not involved in the studio. At the end, a total of 123 questionnaires were entered the study (accurately represented in Table 1), which makes more than 50% of the total number of employees in the Niš Emergency Medical Service, who were eligible to enter the study for 2018.

**Table 1.** Socio-demographic characteristics of respondents-Institute for emergency medicine in Niš – Niš, Republic of Serbia, 2018.

Variables		N	n1	n2
Gender(%)	Male	50 (40.7)	22 (44)	28 (38.40)
	Female	73 (59.3)	28 (56)	45 (61.60)
Age		48.23 ± 8.11	49.80 ± 7.90	47.10 ± 8.10
Occupation (%)	Physician	43 (34.90)	19 (38)	24 (32.88)
	Nurse/technician	48 (39)	14 (28)	34 (46.57)
	Driver	26 (21.10)	12 (24)	14 (19.18)
	Cleaner	6 (4.90)	5 (10)	1 (1.37)
Type of Workplace N (%)	Field work	94 (76.4)	35 (70)	59 (81)
	Ambulance	29 (23.60)	15 (30)	14 (19)
Work in Shifts N (%)	Morning and evening	13 (10.6)	9 (18)	4 (5.5)
	Day and night	110 (89.4)	41 (82)	69 (94.5)
Years of Service (M ± SD)		21.28 ± 9.13	21.20 ± 9.60	21.30 ± 8.80

**Legend:** N = number of all respondents, n1 = Group without exposure, n2 = Group with exposure, M = Mean; SD = Standard deviation.

Representativeness affects the impossibility of generalizing the results, but even as they are, they are significant at the local level in the field of occupational safety of doctors, medical technicians, auxiliary staff and patients as users of medical services, not only at the level of the local community, but also more widely for everyone who rely on the medical services of the test subjects.

### Local

Our research setting was Niš, the third largest city in Serbia and administrative centre of Nišava district, which stretches territory of over 2,700 km^2^. The surface area and the number of inhabitants were the reasons for selecting such research setting^(15)^.

### Selection Criteria

The criteria for inclusion in the research were that respondents are both men and women, employed in the Institute for Emergency Medical Assistance in Niš. In addition, the criterion is that the respondent was exposed to potentially infectious material as part of routine work (blood and other body fluids with possible blood impacts).

### Data Collection

The research was conducted personally by the co-author of the paper, who is also employed in the Institute for Emergency Medical Services in Serbia. The co-author distributed questionnaires to the employees during the break or when changing shifts. Respondents were informed about the goal of the research, signed the consent and we protected their personal data from any kind of abuse.

### Data Analysis and Treatment

For data analysis and processing we used IBM SPSS version 23.0 and G* Power 3.1.9.7^(14)^. Three levels of analysis were performed. First, by obtaining descriptive statistics we got insight in respondents’ profile, their experience and attitudes toward researched topics. Second, differences between groups of main interest were spotted by using t-test and Fisher’s exact test, depending on the nature of researched variables. Finally, binary logistic regression was employed to in order to establish independent risk factors for professional exposure to blood-borne infections of employees. The regression model comprised five independent variables: gender of the respondents, contact with the patient’s blood through the skin, accidental prick on a used needle, occupation and years of service.

### Ethical Aspects

The study was approved by the Ethics Board of the Institute for Emergency Medical Services in Niš (54/2018). All patients provided their Signed Informed Consent Forms before the trial. This study was registered at the Institute for Emergency Medical Services in Niš Registry (http://www.hitnanis.org/) with the registration number (Number 3725). The research used the original questionnaire developed by Predrag Djurić from the Institute of Public Health of Vojvodina, Serbia^(16)^. We obtained author’s approval for questionnaire usage.

## RESULTS

### Respondents’ Profile, Group Differences and Power Size

The research was carried out as a Cross-Sectional Study. In the period from August to October 2018, all healthcare workers working in the Institute for Emergency Medical Services in Niš were invited to participate in the study. The opinion poll encompassed 247 health care workers (the 103 response rate was 49%). Participation in the study was voluntary and anonymous. Each HCW was informed about the purpose of the study and signed an informed consent form.

The response rate in our survey was 49.79%, for a population consisting of 247 respondents. It may be qualified as satisfactory considering average response rates in health related surveys reported in the literature^(17)^. As it is presented in Table 1, all respondents were divided on the two groups in relation to exposure to blood-borne viral infections as key criteria for difference spotting in our research. The first group consisted of 50 (40.7%) respondents without exposure to blood-borne infections and body fluids, while the other group included 73 (59.3%) respondents that are exposed to blood-borne infections and body fluids. A total of 43 physicians, 48 nurses and technicians, 26 drivers and 6 cleaners took part in our survey. Average respondent’s age was 48.23 years. There were 40.7% of male and 59.3% of female respondents. The calculated average year of service was 21.28 years. Statistically significant difference based on all variable was not found between spotted groups drawn on the result (Table 1). With a given groups sizes of n1 = 50 and n2 = 73, and the effect size set at d = 0.50, a compromise power analysis was performed for two-tailed Mann-Whitney U-test and α = 0.012 was calculated. Due to results of the statistical power analysis and multiple comparisons, α level is set to 0.01.

### Descriptive Statistics

Descriptive statistics of our study were presented in Table 2. When the methods of exposure of all respondents to blood and body fluids were analysed, it was indicated that over half of the respondents, more precisely 51.20% reported contact with the blood of patients through the skin. All exposure of all respondents are presented in Table 2.

**Table 2.** Modes and frequencies of accidental events-Institute for emergency medicine in Niš – Niš, Republic of Serbia, 2018.

Modes of professional exposure	Frequency of professional exposure			
	One episode N (%)	Two episodes N (%)	Three or more episodes N (%)	Total N (%)
Accidental stick with used needle	11 (8.90)	2 (1.60)	2 (1.60)	15 (12.20)
Injuries on sharp object	8 (6.50)	4 (3.30)	1 (0.80)	13 (10.60)
Contact with patient’s blood through the skin	14 (11.40)	16 (13)	33 (23.60)	63 (51.20)
Sprayed by patients’ blood on mucous membrane of the eye, nose and mouth	5 (4.10)	3 (2.40)	5 (4.10)	10 (8.10)

**Legend:** N = number of respondents.

Results of risk perception, virus testing and HBV vaccination status of respondents were presented in Table 3.

**Table 3.** Differences between group without and group with exposure-Institute for emergency medicine in Niš – Niš, Republic of Serbia, 2018.

Variables		All respondents, n = 123 (%)	Group without exposure, n1= 50 (%)	Group with exposure, n2 = 73 (%)	χ2, p
Risk perception (exposure to HIV, hepatitis B, and hepatitis C)	yes	120 (97.60)	49 (98)	71 (97.30)	χ^2^ (1) = 2.773, p = 0.96
	no	3 (2.40)	1 (2)	2 (2.70)	
Interventions that bring in contact with blood and other body fluids	yes	114 (92.70)	44 (88)	70 (95.90)	χ^2^ (1) = 2.000, p = 0.16
	no	9 (7.30)	6 (12)	3 (4.10)	
Precautions in working with patients	yes	101 (82.10)	40 (80)	61 (83.60)	χ^2^ (1) = 0.710, p = 0.79
	no	22 (17.90)	10 (20)	12 (16.40)	
Precautions in working with already infected patients	yes	70 (56.90)	30 (60)	40 (54.80)	χ^2^ (1) = 0.150, p = 0.69
	no	53 (43.10)	20 (40)	33 (45.20)	
Testing for HIV	yes	27 (22)	8 (16)	19 (26)	χ^2^ (1) = 1.205, p = 0.27
	no	96 (78)	42 (84)	54 (74)	
Testing for HbcAg	yes	20 (16.30)	6 (12)	14 (19.20)	χ^2^ (1) = 0.658, p = 0.42
	no	103 (83.70)	44 (88)	59 (80.80)	
Testing for Anti-HCV	yes	21 (17.10)	7 (14)	14 (19.20)	χ^2^ (1) = 0.256, p = 0.61
	no	102 (82.90)	43 (86)	59 (80.80)	
Vaccination for hepatitis B	complete	13 (10.60)	4 (8)	9 (12.30)	χ^2^ (4) = 5.021 p = 0.15
	incomplete	8 (6.50)	6 (12)	2 (2.70)	
	no	102 (82.90)	40 (80)	62 (84.90)	

**Legend:** N = number of all respondents, n1 = Group without exposure, n2 = Group with exposure, *χ^2^ the value of the Chi-squared tests, p = statistical significance of differences between groups at the level of p < 0.05.

Considering the perception of risk, 97.60% of the respondents stated that they are at risk of contracting HIV, hepatitis B and hepatitis C infection in the workplace. A total of 92.70% of respondents are performing interventions in which they are in contact with the blood or other body fluids of the patient. In addition, majority of respondents, i.e. 82.10% of respondents, stated that they are taking precautionary measures with each patient in order to protect themselves against blood-borne infections. Majority of respondents, 56.90%, said that they are taking precautionary measures in dealing with patients when there is knowledge that they are infected with HIV, hepatitis B and C, to protect them against blood-borne infections. However, at the same time, 43.10% of respondents stated that they did not implement the above precautionary measures.

In relation to HIV testing, 78% of respondents were not tested for HIV. At the same time in the reference to HbsAg testing, 83.70% of respondents were not tested. With respect to HCV testing, 82.90% of respondents were not tested. In the whole sample, complete vaccination against hepatitis B was reported and showed that 82.90% of respondents were not vaccinated. There were no statistically significant differences between spotted groups of respondent regarding previously mentioned eight questioned parameters at the level of p < 0.050.

### Results of Logistic Regression

The result of multiple binary logistic regression, presented in the Table 4, showed that only three variables gave a unique contribution to the model as risk factors for professional exposure to blood-borne viral infections.

**Table 4.** Predictors of accidental events-Institute for emergency medicine in Niš – Niš, Republic of Serbia, 2018.

Variables	B	S.E.	Odds Ratios	95%CI of Odds Ratios	p*
Constant	–1.30	0.95	0.27	/	0.17
Gender	–0.64	0.66	0.53	0.15–1.92	0.33
Occupation	–0.33	0.64	0.61	0.07–2.51	0.61
Years of service	–0.08	0.04	0.92	0.86–0.10	0.04
Accidental stick on the used needle	4.50	1.19	90.34	8.79–928.03	0.00
Contact to the patients’ blood through the skin	5.18	0.10	176.93	24.95–1254.61	0.00

**Note:** *statistically significant at the level of p < 0.05.

The strongest predictors are accidental used needle stick injuries (OR = 90.34; 95% CI, 8.79–928.03) and contact with the blood of patients through the skin (OR = 176.94; 95% CI, 24.95–1254.61). As a predictor, the years of service are also statistically significant (OR = 0.92; 95% CI, 0.86–1.00).

## DISCUSSIONS

This study represent first attempt to empirically research attitudes toward risk factors of viral infections transmitted by blood-borne pathogens in health institution in Nišava district. It supplemented available knowledge with new data about the frequency of healthcare professionals’ exposure to blood-borne viral infections (HIV, HCV and HBV), as well as the present risk factors. In Serbia, similar studies that were carried out in 2008 and 2016, but only in northern part of country, demonstrated that blood-borne infections with transmissible viruses among healthcare workers continue to be present and that two thirds of viral infections in professional environment comprised of viral hepatitis, hepatitis B virus52% and hepatitis C virus 15%^(16,18)^. Our results show that more than one half of the participants, 97% (71/123) reported exposure incident during the total years of work, and 95% (70/123) that they had contact with blood or other bodily secretions, which is more than in other studies in Serbia where 90% of participants reported exposure to the blood of removable infections^(16)^ and 80% that they had at least one incident in their total work^(18)^. In addition, this study showed a significantly lower testing rate of HBV, HCV and HIV, compared to previous studies conducted in Serbia, 58%^(18)^ and 89% HBV, 91% HCV, 92% HIV in Bosnia and Herzegovina^(19)^, and high level of non-vaccination in our study 83%, and in other study 44%^(16)^, 56% in Serbia^(18)^. Recent studies show that in the whole sample the complete vaccination against hepatitis B was reported by only 20–20.40% of respondents, despite awareness of blood-borne viral infections and effectiveness of vaccination^(20,21,22)^. In high-income countries, the hepatitis B vaccination rate ranges from 42.4% to 86.4%^(23)^. On the other side, our study showed that the work experience is a significant predictor for exposure to blood-borne infection (OR = 0.92; 95% CI, 0.86–1.00), which makes sense, as experienced staff makes fewer mistakes^(24)^. Our results are in line with these results and showed low incidence of occasional situations, but with the years of service, one of the contributing factors was performing work activities in a hurry which should not be happening after years of experience^(24)^. The subjects that were mostly exposed to blood-borne viral infections in our study were nurses/technicians, which is similar to some of previous research^(25)^. This can be explained by the fact that nurses/technicians perform most of the interventions which require the application of intramuscular and intravenous injection therapy using infusion fluids. Nurses/technicians are the main practitioners in administration and implementation of therapy. In support of these results is the fact that in relation to all employees at the Institute for Emergency Medical Services in Niš, the largest numbers of employees were nurses/technicians. However, our results are in contrast withthe results of some previous studies^(26)^, where most accidents (55%) were reported by the doctors. Recently conducted studies in Bosnia and Herzegovina^(19)^, Serbia^(26)^ and China^(27)^ show that many studies that investigated incidents of blood exposures showed some significant differences in prevalence between different professional groups. As an example a study in China in the period from 2015–2018 year proved that nurses are in greater risk of exposure of blood-transfer infections^(28)^, while other studies in Serbia, India and Jamaica proved a greater risk of exposure among physicians^(26,29,30)^. Also, the profile of the nurses/technicians did not prove to be a predictor for the occurrence of professional exposure to blood and body fluids transmissible infections. It should not be forgotten that according to general information there is insufficient data on the profiles of nurses and the organization of jobs that may adversely affect the way they are deployed in the work based on their different skills and level of education. All this resulted in inadequate professional services and low quality of nursing care.

The importance of this study can be reflected in the fact that, so far, little has been done to research the risk factors of blood-borne viral infections in Serbia or among healthcare professionals in emergency medical care facilities. Even though this study has primarily been conducted to point out the importance of exposure to blood-borne viral infections (HIV, HCV and HBV) and their risk factors to all healthcare professionals, the evidence can also help the Ministry of Health to create more advanced health policies and reforms, such as mandatory blood tests for viruses every six months to 1 year, as well as organise and conduct the legal regulations for prevention and observation of the immunological status of healthcare professionals, exposure control and protective measures.

Although the statistical relevance of some of the variables was not established, they confirmed that they exist in the studied population (such as the non-vaccination of the medical staff or the non-testing of them), which indicates the need to invest efforts in the educational field, increased control of the work of employees, while providing resources for protection at work (gloves, protective masks, means of maintaining the hygiene of space and people). The evidence gathered can further serve to the healthcare professionals and decision-makers, to not only protect healthcare professionals, but also the entire population. An unvaccinated and untested healthcare professional presents a danger to the citizens who require medical aid. For many years there have been no healthcare reforms in Serbia. So these guidelines can change the practice and contribute to solving the issues of the exposure of healthcare professionals and the risk factors for blood-borne viral infections, as well as to improve the final outcome for the patients.

## CONCLUSIONS

More than half of the respondents reported accident events at their workplace that have been treated as risk factors for blood-borne viral infections. At the same time, despite awareness of infections there is low level of vaccinated staff. Differences in attitudes between group of respondents without exposure and group with exposure were not found.

Also, the limitations of this study are reflected in the lack of results of testing employees for blood-borne diseases, which was not conducted due to financial reasons and poor response of employees.

The limitation of this study is that no one has conducted an exact same study in other Institutes for Emergency Medical Services in Serbia therefore the results of this study are impossible to compare to other studies. In that sense, it is necessary to conduct a study of this kind in other Institutes for Emergency Medical Services in Serbia.

The results of this study indicate the necessity of data-based planning of prevention of professional infectious diseases, and ensuring a healthy and safe working environment in the sense of eliminating or reducing the likelihood of exposure and risk factors.

Finally, the special importance of this study, both at the local and international level, is reflected in the indication of the high risk of infection in the field of emergency medical care and the increased need for education and control of the work of employees, while providing resources for protection at work (gloves, protective masks), means of maintaining the hygiene of space and people).
